# Intranasal Seletracetam in a Patient with Reading Epilepsy: First‐in‐Human Use to Prevent Reflex Seizures

**DOI:** 10.1002/ana.78128

**Published:** 2025-12-29

**Authors:** Matthias J. Koepp, Kai‐Nicolas Poppert, Thomas Felder, Aljoscha Thomschewski, Sandra Lafenthaler, Pavel Klein, Alexander Rotenberg, Wolfgang Löscher, Chris Rundfeldt, Eugen Trinka

**Affiliations:** ^1^ Department of Neurology National Hospital for Neurology and Neurosurgery, Queens Square London UK; ^2^ Department of Clinical and Experimental Epilepsy, UCL Queen Square Institute of Neurology University College London London UK; ^3^ Department of Neurology Neurocritical Care and Neurorehabilitation, Christian Doppler University Hospital, Centre of Cognitive Neuroscience, Paracelsus Medical University, Salzburg, Member of EpiCARE Salzburg Austria; ^4^ Department of Laboratory Medicine Paracelsus Medical University Salzburg Austria; ^5^ Neuroscience Institute, Christian Doppler University Hospital, Centre of Cognitive Neuroscience, Paracelsus Medical University Salzburg Austria; ^6^ Mid‐Atlantic Epilepsy and Sleep Center Bethesda MD USA; ^7^ Department of Neurology Boston Children's Hospital and Harvard Medical School Boston MA USA; ^8^ Translational Neuropharmacology Lab, NIFE, Department of Experimental Otology of the ENT Clinics, Hannover Medical School Hannover Germany; ^9^ Drug Consulting Network Coswig Germany; ^10^ Karl Landsteiner Institute for Clinical Neuroscience Salzburg Austria

## Abstract

We report the first human use of intranasal seletracetam (SEL) to prevent reflex seizures. A patient with epilepsy with reading‐induced seizures on levetiracetam (3,000 mg/day) continued to experience reading‐induced focal seizures with preserved consciousness. Detectable in serum within 2 minutes of intranasal administration, 30 mg seletracetam delayed seizure onset from 1:56 (placebo) to 4:17 minutes post‐stimulus onset. A second 30 mg dose fully prevented seizures during 25 minutes of reading. Electroencephalogram (EEG) spike‐frequency declined dose‐dependently (3.1/min at placebo to 1.6/min after second dose), with reduced spike‐propagation on magnetoencephalography (MEG). Our findings support SEL as a promising non‐benzodiazepine acute seizure prevention and provide insight into reflex seizure dynamics. ANN NEUROL 2026;99:535–539

Acute repetitive seizures (ARS), or seizure clusters, affect many patients with epilepsy and are linked to increased morbidity, emergency care use, hospitalizations, and socio‐economic burden.^1^ Current ARS rescue therapies rely on benzodiazepines, which, although effective, carry risks of sedation, respiratory depression, and dependency.^2^, ^3^ Due to these risks, the US Food and Drug Administration (FDA) restricts intranasal benzodiazepine use to one dose every 3 to 5 days and no more than 5 doses monthly highlighting the need for non‐benzodiazepine alternatives.^4^, ^5^

Seletracetam (SEL), a potent antiseizure medication (ASM) targeting SV2A, is ~ 100 times more potent in blocking seizures than levetiracetam (LEV) and ~10 times more potent than brivaracetam (BRV) in animal models.^6^ Initially developed by UCB Pharma through phase 2a trials as a back‐up to BRV, SEL showed favorable efficacy and safety after oral use but was halted in development. Still, SEL remains one of the most potent compounds tested in animal models and the human photosensitivity model, with clinical potential.^7^ Its potency and high water solubility allow delivery in the small liquid volume (100–150 μl) required for intranasal use. Its rapid membrane penetration makes SEL a strong candidate as the first non‐benzodiazepine intranasal rescue therapy for ARS.^8^

In reflex epilepsies, seizures are triggered by stimuli like flashing lights or reading.^9^, ^10^ Epilepsy with reading‐induced seizures (EwRIS) offers a controlled model to test ARS interventions. We report the first case of intranasal SEL in a patient with EwRIS and focal seizures with preserved consciousness localizing to the premotor cortex via electroencephalogram (EEG), functional magnetic resonance imaging (fMRI), and magnetoencephalography MEG.^11^ To complement seizure observation and subjective self‐reporting of sensory seizures, we used EEG and MEG to objectively measure seizure activity and brain network dynamics in response to intranasal SEL.

## Methods

A 42‐year‐old man with EwRIS^11^ experienced predictable reading‐induced focal seizures with preserved consciousness – jaw‐clicking sensations and, if reading continued, oro‐facial myocloni. These previously led to focal‐to‐bilateral tonic–clonic seizures before high‐dose LEV (3,000 mg/day). He gave informed consent, and approval was granted by the institution (Christian Doppler University Hospital Salzburg) to treatment with intranasal seletracetam under Austria's “Named Patient Use” regulation (Article 83 of Regulation 726/2004/EC, § 8 Abs. 1 Z 2 Austrian Medicinal Products Act 2009), allowing drug use outside formal development programs. In Austria, the conduct of such a therapeutic trial is not subject to approval by Federal State Ethics Commission, or notification to the Austrian Medicines Agency, but is the sole responsibility of the treating physician.

Intranasal SEL (99.88% purity; MedChem Express, Monmouth Junction, NJ) was prepared per protocol by Federal Pharmacy Salzburg (“Landesapotheke Salzburg”), ensuring identity, strength, quality, and purity. The 30 mg of SEL was diluted in 150 μl potassium phosphate‐buffered 0.75% hydroxyethyl cellulose (Natrosol) solution (pH 7.0). Cellulose enhanced mucosal adherence and reduced mucociliary clearance. Then, 150 μl volumes were delivered into one nostril via syringe with a special nasal spray adaptor.^8^

After confirming that reading reliably triggered 3 seizures, saline placebo (0.9% NaCl, 100 μl) was first administered to acclimate the patient to nasal delivery. Upon seizure recurrence, a 30 mg SEL dose was given; a second was delivered if seizures recurred.

**FIGURE 1:**
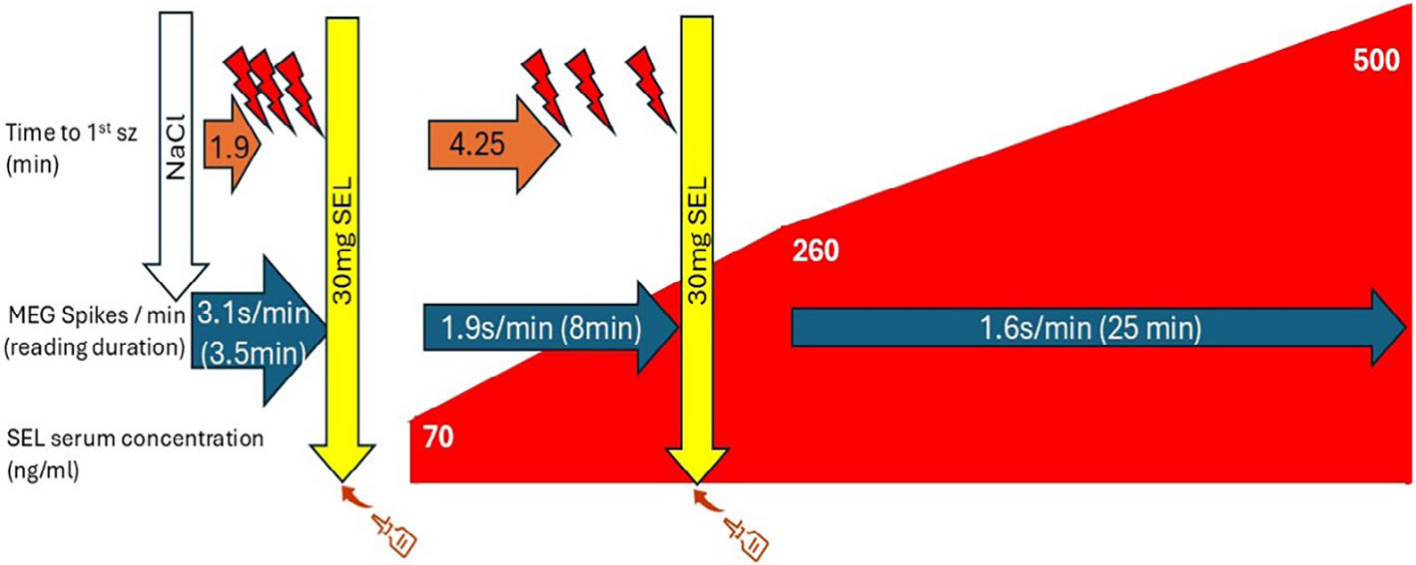
Flow diagram of protocol. After saline treatment, the first reading‐induced seizure occurred after 1:56 minutes (1.9 minutes) of reading. Following the third unequivocal seizure, intranasal SEL (30 mg) was administered, which delayed seizure onset to 4:17 minutes (4.25 minutes; length of the *orange arrow* is proportional to the time to the first seizure). Following a further 2 unequivocal seizures and a second 30 mg dose, no further seizures occurred despite continuing to read the same material for a further 25 minutes. MEG spike frequency was 3.1/min after saline, declining to 1.9/min after the first, and to 1.6/min after the second 30 mg SEL doses (length of the *blue arrows* is proportional to the duration of reading, and the thickness of the *arrows* is proportional to the frequency of reading‐induced spikes). SEL serum concentrations were measured 2:38 minutes after the first SEL dose, 2:58 minutes after the second SEL dose, and again after ~25 minutes of reading, or 40:37 minutes after the first SEL dose. SEL serum levels were 70 ng/ml after the first SEL dose, 260 ng/ml after the second SEL dose, and 500 ng/ml at the end of the study (the area covered in *red* is proportional to the measured increase in SEL serum levels). MEG = magnetoencephalography; SEL = seletracetam. [Color figure can be viewed at www.annalsofneurology.org]

Serum samples were collected ~2 minutes after placebo and each SEL dose, and again ~30 minutes post‐second dose (Fig 1). SEL levels were measured via high‐performance liquid‐chromatography tandem mass spectrometry (HPLC‐MS/MS).

MEG was recorded with a 306‐channel Elekta‐Neuromag system (Helsinki, Finland), with reading stimuli projected. EEG was simultaneously recorded using a 64‐channel Elekta cap. MEG and EEG data were visually inspected; interictal spikes were selected and source localization done using Brainstorm software.^12^

## Results

Baseline recording identified selected passages of mathematical lecture notes^13^ as the most reliable seizure‐provoking text, capturing 9 spikes at a rate of 1.3/minute during reading; no spikes occurred at rest. A test dose of 150 μl NaCl (placebo) was administered via syringe with a special nasal spray adaptor to prepare the patient for the sensation of intranasal delivery. The first seizure began 1 minute and 56 seconds after reading onset (see Fig 1). Reading stopped after the third clear seizure (~4 minutes in).

The first 30 mg SEL dose was then delivered into the left nostril. After ~2 min, reading resumed. The first seizure post‐SEL was delayed to 4 minutes and 17 seconds. It was subtle, so the patient continued reading. The first unequivocal seizure occurred ~8 minutes in, prompting the second 30 mg SEL dose into the right nostril. Three minutes later, the patient resumed reading and continued for 25 minutes without any self‐reported seizures—a > 10× increase in seizure‐free reading time.

MEG recording continued throughout, up to 30 minutes post‐second SEL dose. Left frontal spikes decreased from 3.1/min (baseline, after NaCl) to 1.9/min (after first SEL) and 1.6/min (after second SEL).

The only adverse effect was a mild, transient bitter taste after the first dose, likely from partial swallowing. No sedation was observed.

After the first dose, plasma SEL reached 70 ng/ml (301 nM), rising to 260 ng/ml (1,120 nM) ~3 minutes after the second dose, and 500 ng/ml (2,153 nM) at 27:15 minutes.

**Figure 2:**
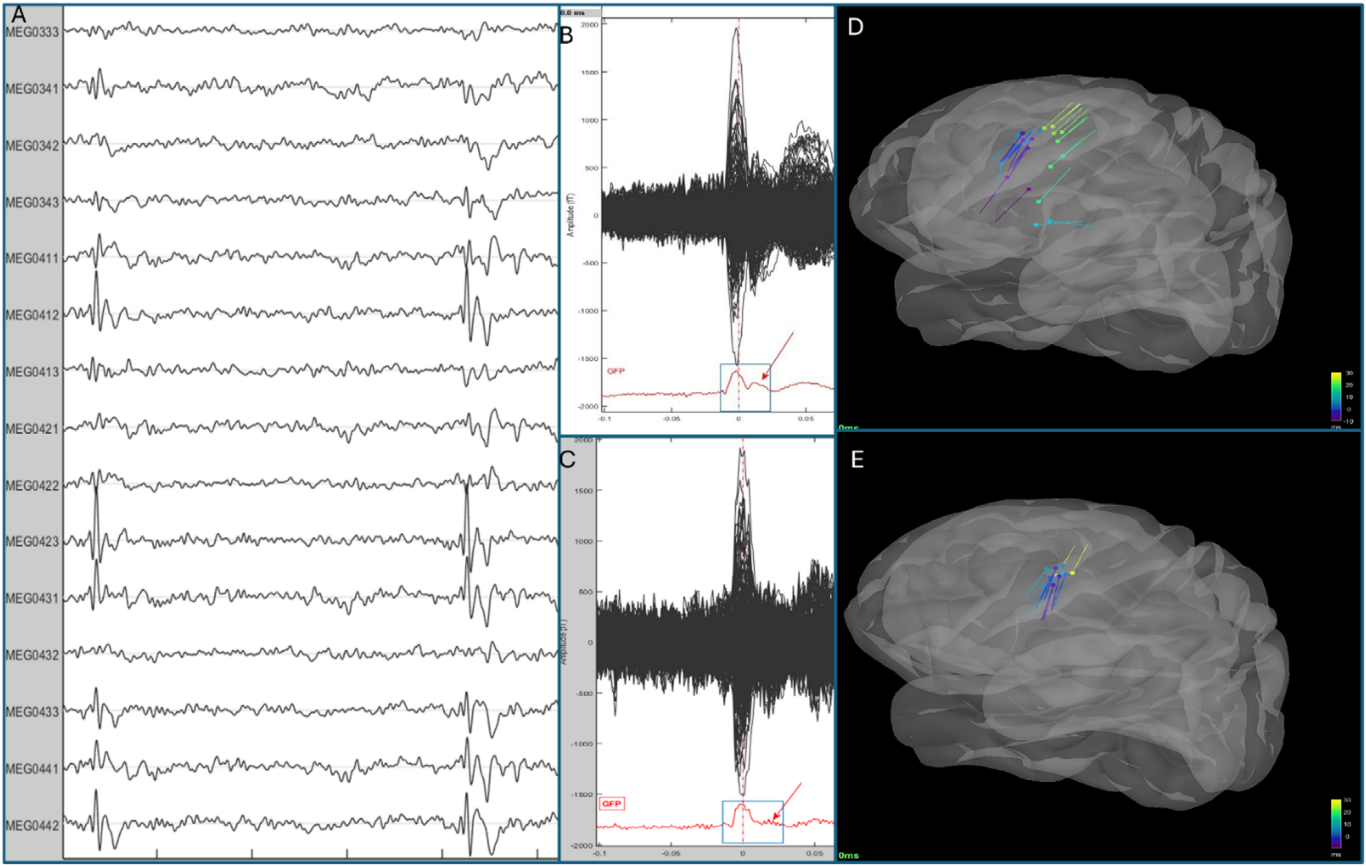
MEG analysis: changes in spike propagation. The left panel (A) is representative of the MEG epoch containing 2 epileptic spikes. The middle panel (B, C) shows the Butterfly plots displaying spike‐averaged data from the placebo phase (B) and after the second SEL administration (C). GFPs are shown at the bottom of each plot. A clear difference in GFP morphology is evident between conditions (*red arrows*): during the placebo phase (B), a prominent secondary peak appears immediately following the primary spike maximum, which is markedly reduced after SEL treatment (C). The right panel (D, E) shows the results of dipole scanning performed over a time window from −10 ms to +30 ms relative to the spike maximum, visualized on a 3D brain model (Goodness of fit = 60%). The spatial distribution of peak dipole activations demonstrates broader cortical involvement during the placebo condition (D), with extension toward the ipsilateral insular region, compared to the more restricted activation pattern following the second SEL administration (E). This spatial expansion temporally corresponds to the secondary GFP peak observed in the time‐domain analysis. GFPs = global field potentials; MEG = magnetoencephalography; SEL = seletracetam. The figure was created using Brainstorm.^12^ [Color figure can be viewed at www.annalsofneurology.org]

Spike propagation and frequency decreased post‐SEL. Dipole modeling of averaged spikes showed initial activity in the left precentral area (mouth motor cortex), consistent with prior studies.^11^ Before SEL, spikes propagated to the left posterior insula. After the second dose, activity remained localized, consistent with seizure cessation (Fig 2).

## Discussion

In this Named Patient Use case, 60 mg of intranasal SEL prevented reading‐induced seizures. The first 30 mg dose delayed seizure onset; the second fully blocked seizures. MEG spike frequency and propagation decreased, supporting the clinical findings.

Intranasal SEL was well tolerated. A bitter taste after the first dose likely indicated some swallowing, possibly delaying efficacy; this was absent after the second dose. SEL serum levels rose rapidly, showing fast intranasal absorption. Although sampling was limited, levels resembled those from oral dosing, where 50 mg produced ~1,000 ng/ml within an hour in healthy volunteers.^14^ The concentration range (0.3–2.15 μM) of SEL matched levels that suppressed epileptiform activity in rat hippocampal slices,^15^ consistent with clinical seizure termination.

Given that SEL is < 10% protein‐bound, these concentrations likely reflect relevant brain exposure.^15^ The pharmacokinetic and anti‐seizure profiles align with unpublished phase 2a studies showing SEL efficacy at 10 to 80 mg orally twice daily (NCT00152503 and NCT00152451).

Although intranasal and buccal benzodiazepines are established rescue treatments, their use is limited by risks of sedation, respiratory depression, and addiction.^4^, ^5^ Intranasal SEL offers a fast‐acting, non‐sedating alternative without known dependency risk. Unlike diazepam (*t*½ ~30 hours) and desmethyldiazepam (*t*½ up to 200 hours),^16^ SEL has a short plasma half‐life (~8 hours), rapid absorption, and no active metabolites, reducing the risk of accumulation with repeated use.^14^

In phase I and 2 phase IIa trials (11 weeks) in patients with drug‐resistant focal‐onset seizures, no withdrawal signs were observed after stopping SEL. Preclinical studies also showed no addictive potential.^14^ This aligns with other racetams like LEV (no appreciable abuse risk) and BRV (very low abuse potential), distinguishing them from benzodiazepines.

These properties make SEL particularly suitable for acute, intermittent use in reflex epilepsies avoiding complications of chronic benzodiazepine therapy. Its pharmacological profile supports its potential as a rescue therapy for ARS.

Effective ARS treatment reduces risk and provides reassurance during triggers—such as reading—for patients with reflex epilepsy, like ours, who previously experienced focal‐to‐bilateral tonic–clonic seizures before high‐dose LEV.

Indeed, intranasal SEL meets key ARS treatment criteria:

- Rapid seizure termination: The second 30 mg SEL dose prevented seizures that previously occurred in under 2 minutes after placebo.
- Ease of administration: Intranasal delivery was practical and noninvasive, although partial swallowing may affect initial dose effectiveness.
- Safe repeated dosing: Two doses were well tolerated, with no sedation. SEL was tolerated orally in phase 1 trials at up to 600 mg, 10× the effective dose used here.^14^

In addition to patient‐reported improvement, we used objective MEG and EEG measures to confirm that SEL rapidly reduced spike frequency and cerebral excitability. This suppression of subjective and electrophysiologic correlates is unlikely due to habituation or stimulus exhaustion, as continuous reading reliably triggered spikes and seizures earlier that day and up to 150/hour during prior EEG‐fMRI studies.^11^ The reading material was linguistically complex and unpredictable, minimizing the chance of neural adaptation.

Spike propagation became more confined to the initial seizure onset zone after SEL, whereas placebo allowed spread to the ipsilateral insula. SEL reduced both spike frequency and spatial spread, aligning with seizure control. This supports a network‐stabilizing effect, consistent with evidence that effective treatments limit both frequency and spatial dynamics of epileptic activity.^17^

After SEL, the patient read for 25 minutes without seizures—over 10‐fold longer than any seizure‐free interval earlier that day. Based on the “rule of 3,”^18^ this sustained suppression cannot be attributed to habituation but to intranasal SEL.

SEL represents a promising, non‐sedating, non‐benzodiazepine treatment for ARS, demonstrating rapid and effective seizure prevention in reading‐induced epilepsy, supported by objective EEG/MEG evidence of reduced spike frequency and restricted propagation.

## Author Contributions

M.K., P.K., C.R., W.L., A.R., and E.T. contributed to the conception and design of the study; M.K., K.P., T.F., A.T., S.L. and E.T. contributed to the acquisition and analysis of data; M.K., K.P., T.F., A.T., S.L. and E.T. contributed to drafting the text or preparing the figures. [Correction added on 09 February 2026, after first online publication: Author contribution text has been revised in this version.]

## Potential Conflicts of Interest

M.K., P.K., and E.T. have received honoraria, travel support and research funding from UCB, which has originally developed seletracetam. M.K., P.K., A.R., W.L., C.R., and E.T. are co‐founders of PrevEp, Inc. which is involved in the development of an intranasal formulation of seletracetam. The remaining authors have nothing to report.

## Data Availability

The data that support the findings of this study are available on request from the corresponding author. The data are not publicly available due to privacy or ethical restrictions.
